# A statistical model for reference-free inference of archaic local ancestry

**DOI:** 10.1371/journal.pgen.1008175

**Published:** 2019-05-28

**Authors:** Arun Durvasula, Sriram Sankararaman

**Affiliations:** 1 Department of Human Genetics, David Geffen School of Medicine, University of California, Los Angeles, Los Angeles, California; 2 Department of Computer Science, University of California, Los Angeles, Los Angeles, California; 3 Bioinformatics Interdepartmental Program, University of California, Los Angeles, Los Angeles, California; 4 Department of Computational Medicine, University of California, Los Angeles, Los Angeles, California; University of Washington, UNITED STATES

## Abstract

Statistical analyses of genomic data from diverse human populations have demonstrated that archaic hominins, such as Neanderthals and Denisovans, interbred or admixed with the ancestors of present-day humans. Central to these analyses are methods for inferring archaic ancestry along the genomes of present-day individuals (*archaic local ancestry*). Methods for archaic local ancestry inference rely on the availability of reference genomes from the ancestral archaic populations for accurate inference. However, several instances of archaic admixture lack reference archaic genomes, making it difficult to characterize these events. We present a statistical method that combines diverse population genetic summary statistics to infer archaic local ancestry without access to an archaic reference genome. We validate the accuracy and robustness of our method in simulations. When applied to genomes of European individuals, our method recovers segments that are substantially enriched for Neanderthal ancestry, even though our method did not have access to any Neanderthal reference genomes.

## Author summary

Recent analyses of modern human genomes have shown that archaic hominins like Neanderthals and Denisovans contribute a few percentage of ancestry to many populations. These analyses rely on having accurate reference genomes from these archaic populations. Due to the difficulty in sequencing these genomes, we lack a complete collection of reference genomes with which to identify archaic ancestry. Here, we develop a method that identifies segments of archaic ancestry in modern human genomes without the need for archaic reference genomes. We systematically evaluate the accuracy and robustness of our method and apply it to modern European genomes to uncover signals of introgression which we confirm to be from a population related to Neanderthals.

## Introduction

Admixture, the exchange of genes among previously isolated populations, is increasingly being recognized as an important force in shaping genetic variation in natural populations. Analyses of large collections of genome sequences have shown that admixture events have been prevalent throughout human history [1]. These studies have shown that modern human populations outside of Africa trace a small percentage of their ancestry to admixture events from populations related to archaic hominins like Neanderthals and Denisovans [1, 2, 3]. Further, studies of the functional impact of archaic ancestry have suggested that Neanderthal DNA contributes to phenotypic variation in modern humans [4, 5].

Central to these studies is the problem of archaic local ancestry inference—the pinpointing of segments of an individual genome that trace their ancestry to archaic hominin populations. Methods for archaic local ancestry inference leverage various summary statistics computed from modern and ancient genomes. For example, at a given genomic locus, individuals with archaic ancestry are expected to have low sequence divergence to an archaic genome [6]. A number of summary statistics [7, 8, 9] as well as statistical models that combine these statistics [2, 10, 11, 12] to infer archaic local ancestry have been proposed.

These methods are most effective in settings where reference genomes that represent genetic variation in the archaic population are available. For example, the analyses of Neanderthal [6, 10] and Denisovan admixture events [13] relied on the genome sequences from the respective archaic populations. In a number of instances, however, the archaic population is either unknown or lacks suitable reference genomes. Several recent studies have found evidence for archaic introgression in present-day African populations from an unknown archaic hominin [14, 15, 16] while analysis of the high-coverage Denisovan genome has suggested that the sequenced individual traces a small proportion of its ancestry to a highly-diverged unknown archaic hominin [10].

One of the most widely used statistics for identifying archaic ancestry is the S*-statistic [9], which identifies highly diverged SNPs that are in high linkage disequilibrium (LD) with each other in the present-day population as likely to be introgressed. The S*-statistic is attractive as it can be applied even where no reference genome is available. However, the power of the S*-statistic tends to be low in the reference-free setting [3] and its accuracy depends on a number of parameters that need to be fixed in advance.

Here, we introduce a new statistical method, ARCHaic Introgression Explorer (ArchIE), that combines several population genetic summary statistics to accurately infer archaic local ancestry without the need for a reference genome. ArchIE is based on a logistic regression model that predicts the probability of archaic ancestry for each window along an individual genome. The parameters of ArchIE are estimated from training data generated using coalescent simulations. Our proposed method has several advantages. First, the model can incorporate a variety of statistics that are potentially informative of archaic ancestry. This flexibility allows the model to be applied to the reference-free setting (the setting that is the focus in this paper). However, the model can be extended to also incorporate reference genomes when available, even when these reference genomes might be from distant representatives [10] or from low-coverage samples [17, 18]. Second, our use of a statistical model allows us to efficiently estimate model parameters that optimize desired objective functions such as the likelihood. This property allows the model to be adapted to admixture events with different time depths or admixture fractions as well as to infer other population genetic parameters of interest. Indeed, recent studies have shown that statistical predictors that combine weakly-informative summary statistics can substantially improve a number of population genetic inference problems [19, 20, 21].

We show that ArchIE obtains improved accuracy in simulations over the S*-statistic (as well as the recently proposed S’ method [22]) while being robust to demographic model misspecifications that can cause the distribution of features and archaic ancestry labels in the training data to differ from the test data. We apply ArchIE to Western European (CEU) genomes from the 1000 Genomes project and show that the segments inferred to harbor archaic ancestry have an increased likelihood of being introgressed from Neanderthals even though no Neanderthal genome was used in the inference. These segments recover previously observed features of introgressed Neanderthal ancestry: we observe a decreased frequency of these segments in regions of the genome with stronger selective constraint [23] as well as elevated frequency at the BNC2 and OAS loci that have previously been reported to harbor elevated frequencies of Neanderthal ancestry [2, 3].

## Results

### Overview of statistical model to detect archaic local ancestry

**Fig 1:**
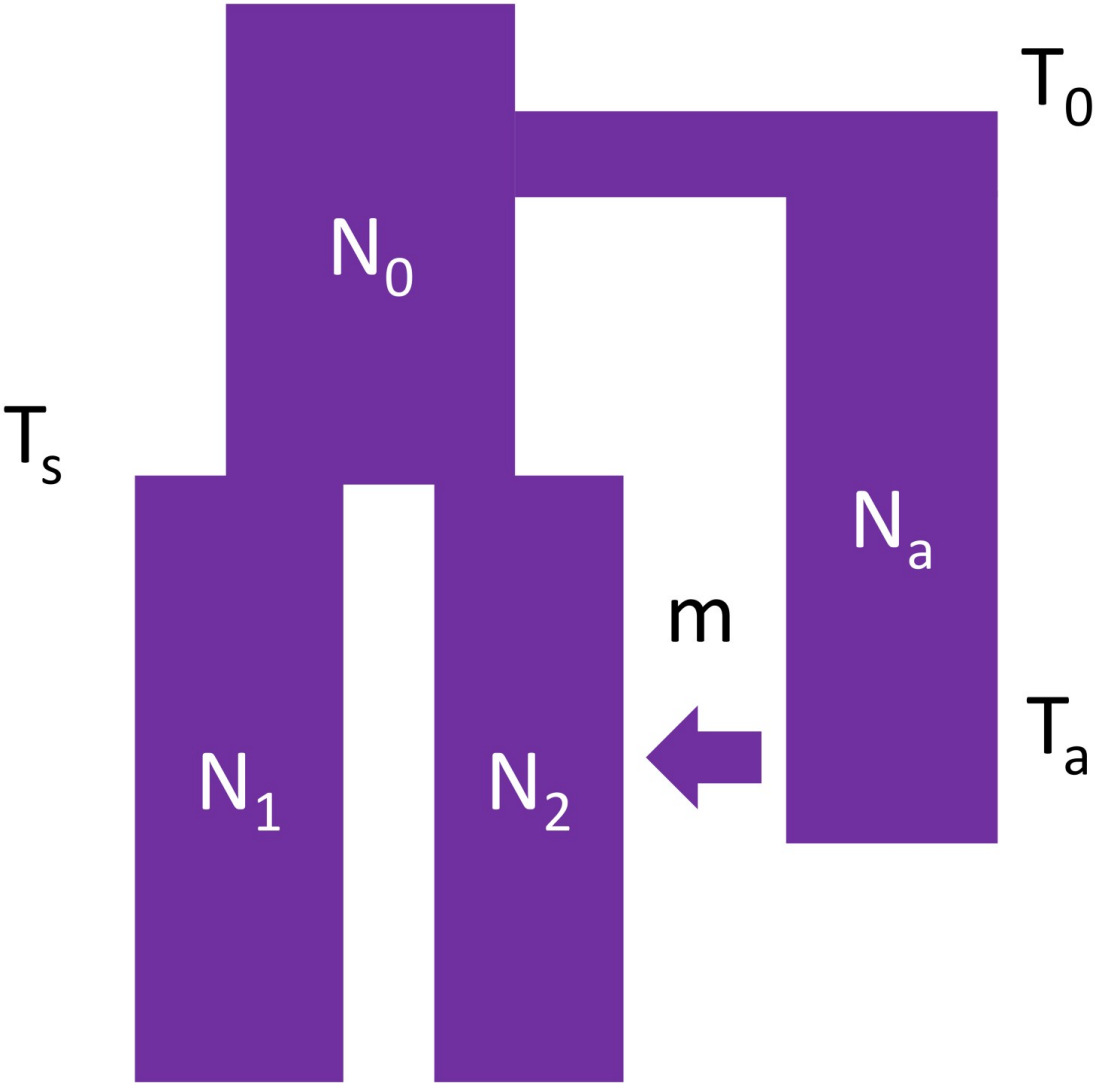
Outline of the demographic model used for training ArchIE. We simulate a population starting at size *N*_0_ and splitting into archaic and modern human (MH) populations at time *T*_0_. The MH population splits into a reference and target population of size *N*_1_ and *N*_2_, respectively, at time *T*_*s*_. Then, at time *T*_*a*_, the archaic population admixes with the target population with an associated admixture proportion *m*. We use data simulated from this model to train a logistic regression classifier.

Our method, ArchIE, aims to predict the archaic local ancestry state in a given window along an individual haploid genome. This prediction is performed using a binary logistic regression model given a set of features computed within this window. Estimating the parameters of this model requires labeled training data *i.e*., a dataset containing pairs of features and the archaic local ancestry state for a given window along an individual genome. To obtain labeled training data, we simulate data under a demographic model that includes archaic introgression, label windows as archaic or not, compute features that are potentially informative of introgression, and estimate the parameters of our predictor on the resulting training data (Fig 1A, Methods). While our method is general enough to be applicable to non-human populations, we describe the demographic model in terms of a modern human-archaic human demographic history.

We simulate training data using a modified version of the coalescent simulator, ms [24], which allows us to track each individual’s ancestry. We use the demographic model from Sankararaman *et al*. 2014 [2] (See Table 1). In this model, an ancestral population splits *T*_0_ generations before present (B.P.) forming two populations (archaic and modern human in the case of the Neanderthal-human demography). The modern human population subsequently splits into two populations *T*_*s*_ generations B.P., one of which then interbreeds with the archaic population (referred to as the target population) while the other does not (the reference population). We simulate one haploid genome (haplotype) in the archaic population, 100 haplotypes in the target population and 100 haplotypes in the reference population (thus, a target population consists of 50 diploid individuals). We sample the archaic haplotype at the same time as the modern human haplotypes, but the statistics we calculate do not rely on features of the archaic genome. We simulate 10,000 replicates of 50,000 base pairs each (bp), resulting in 1,000,000 training examples. We use a window of length 50 Kb because that is the mean length of the introgressed archaic haplotype after *T*_*a*_ = 2, 000 generations based on the recombination rate assumed in our simulations.

**Table 1:** Parameters used in training simulations.

Parameter	Description	Value
*N*_1_	Reference population size	10000
*N*_2_	Target population size	10000
*N*_*a*_	Archaic population size	10000
*N*_0_	Ancestral population size	10000
*m*	Admixture fraction	2%
*T*_0_	Archaic split time	12000
*T*_*S*_	Target-Reference split time	2500
*T*_*a*_	Admixture time	2000
*μ*	Per base pair mutation rate	1.25 x 10^−8^
*r*	Per base pair recombination rate	1 x 10^−8^

We summarize the training data using features that are likely to be informative of archaic admixture. Since we are interested in the probability of archaic ancestry for a given focal haplotype, we compute features that are specific for the focal haplotype. First, for the focal haplotype, we calculate an individual frequency spectrum (IFS), which is a vector of length *n*, the haploid sample size of the target population. Each entry in the vector is the number of mutations on the focal haplotype that are segregating in the target population with a specific count of derived alleles. Due to the accumulation of private mutations in the archaic population, we expect the IFS to capture the excess of alleles segregating at frequencies close to the admixture fraction in the introgressed population. This statistic is closely related to the conditional site frequency spectrum [25].

Next, we calculate the Euclidean distance between the focal haplotype and all other haplotypes, resulting in a vector of length *n*. Under a scenario of archaic admixture, the distribution of pairwise differences is expected to differ when we compare two haplotypes that are both modern human or archaic versus when we compare an archaic haplotype to a modern human haplotype. We also include the first four moments of this distribution, *i.e*., the mean, variance, skew, and kurtosis. These summaries of haplotype distance are similar to the *D*_1_ statistic used in Hammer *et. al*. [14].

The next set of features rely on a present-day reference human population that has a different demographic history compared to the target population. The choice of the reference can alter the specific admixture events that our method is sensitive to: we expect the method to be sensitive to admixture events in the history of the target population since its divergence from the reference. While our method can also be applied in the setting where no such reference population exists, in the context of human populations where genomes from a diverse set of populations is available [1], the use of the reference can improve the accuracy and the interpretability of our predictions. Given a reference population, we compute the minimum distance of the focal haplotype to all haplotypes in the reference population. A larger distance is suggestive of admixture from a population that diverged from the ancestor of the target and reference populations before the reference and target populations split. This feature shares some similarities with the *D*_2_ statistic from Hammer *et. al*. [14].

We also calculate the number of SNPs private to the focal haplotype, removing SNPs shared with the reference, as these SNPs are suggestive of an introgressed haplotype. Finally, we calculate S* [9], a statistic designed for detecting archaic admixture by looking for long stretches of derived alleles in high LD.

Using these features, we train a logistic regression classifier to distinguish between archaic and non archaic segments. In our training data, we define archaic haplotypes as those for which ≥ 70% of bases are truly archaic in ancestry and non-archaic as those for which ≤ 30% are archaic in ancestry. We discard haplotypes that fall in-between those values in the training data resulting in 988,372 training examples.

### Accuracy of estimates of archaic local ancestry

**Fig 2:**
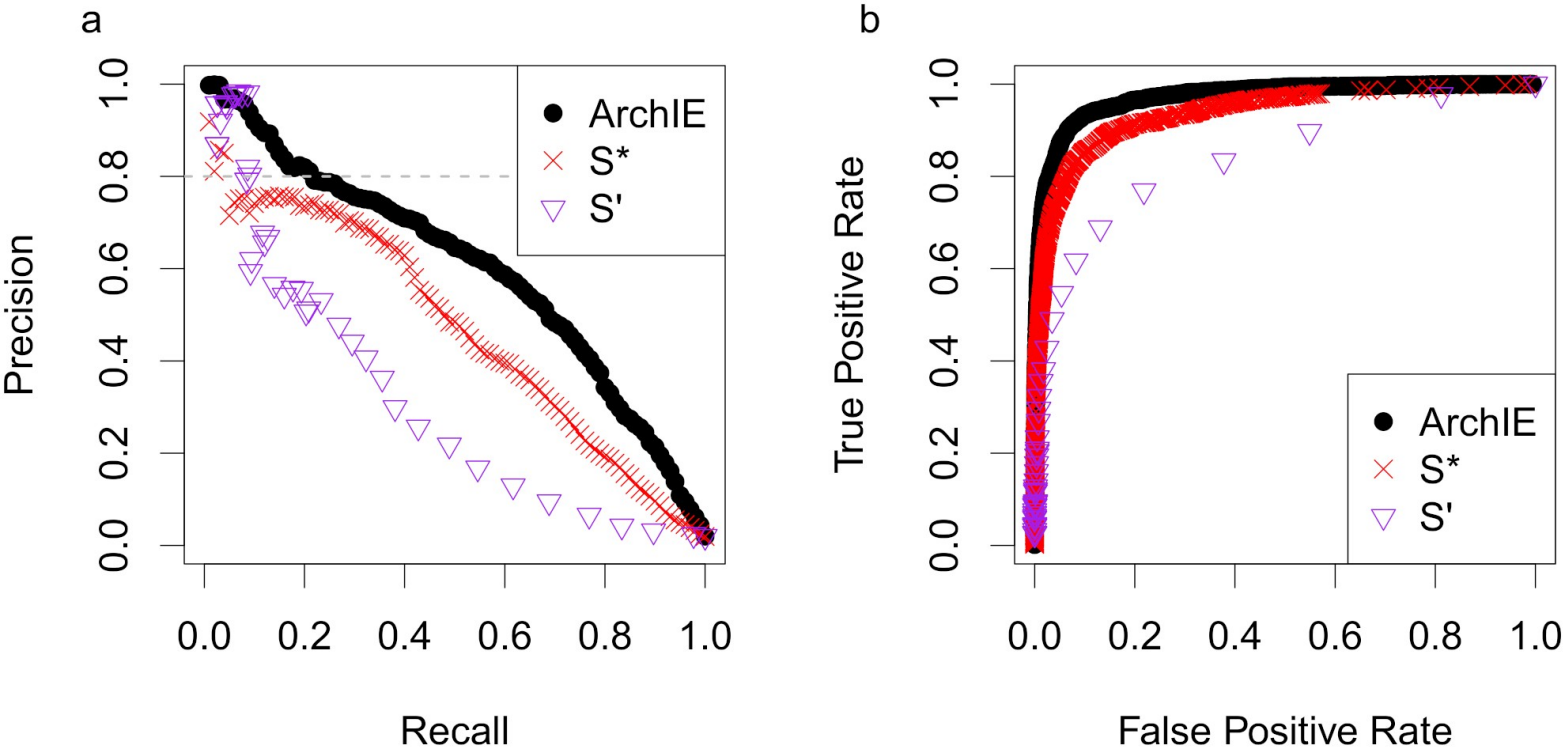
ArchIE obtains improved accuracy over related methods. (A) Precision-Recall (PR) and (B) Receiver Operator Characteristic (ROC) curves for ArchIE (black circles), S* (red crosses), and S’ (purple triangles) in a 2% admixture scenario with a Human-Neanderthal demography. The dashed line corresponds to a false discovery rate of 20%.

We tested the accuracy of ArchIE by simulating data under a demography reflective of the history of Neanderthals and present-day humans [2]. We evaluated the ability of ArchIE to correctly predict the archaic ancestry at each SNP along an individual haplotype. Since ArchIE predicts archaic ancestry within a window, we simulated a 1 Mb segment, applied ArchIE in a 50 Kb window that slides 10 Kb at a time, and predicted archaic ancestry at a SNP by averaging predictions across all windows that overlap the SNP (Methods). We compute Receiver Operator Characteristic (ROC) and Precision Recall (PR) curves by varying the threshold at which we call a SNP archaic and calculating the true positive rate (TPR), false positive rate (FPR), precision, and recall (Fig 2).

We compared ArchIE to an implementation of the S*-statistic from Vernot and Akey using their hyper parameter choices [3] and to S’, a new method for reference-free inference of archaic ancestry [22] (Methods). At a 2% admixture fraction, ArchIE outperforms the S* and S’ statistics across all thresholds (Fig 2A and 2B). At a precision of 0.80, *i.e*., false discovery rate of 20%, ArchIE obtains a recall of 0.21, S* obtains a recall of 0.04, and S’ obtains a recall of 0.09. The area under the ROC curve (AUROC) is 0.94 (±0.008) for S*, 0.84 (±0.01) for S’, and 0.97 (±0.005) for ArchIE and the area under the PR curve (AUPR) is 0.47 for S* (±0.031), 0.28 (±0.032) for S’, and 0.60 (±0.05) for ArchIE (All standard error were estimated using a block jackknife [26] using 1 Mb blocks). We also note that while the ROC curves are similar, the PR curves show a large difference, indicative of the utility of PR curves in problems where there is an imbalance in the frequencies of the two classes.

We also evaluated the ability of ArchIE to call archaic haplotypes. Since haplotypes can range from having none of their ancestry to being entirely from the archaic population, we called haplotypes archaic if they contain ≥ 70% archaic ancestry or not archaic if they contain ≤ 30%. We see that again, ArchIE has larger AUPR (0.53 for ArchIE, 0.38 for S*) and AUROC (0.97 for ArchIE, 0.94 for S*) compared to S* (S4 Fig).

### Population genetic features informative of archaic local ancestry

**Fig 3:**
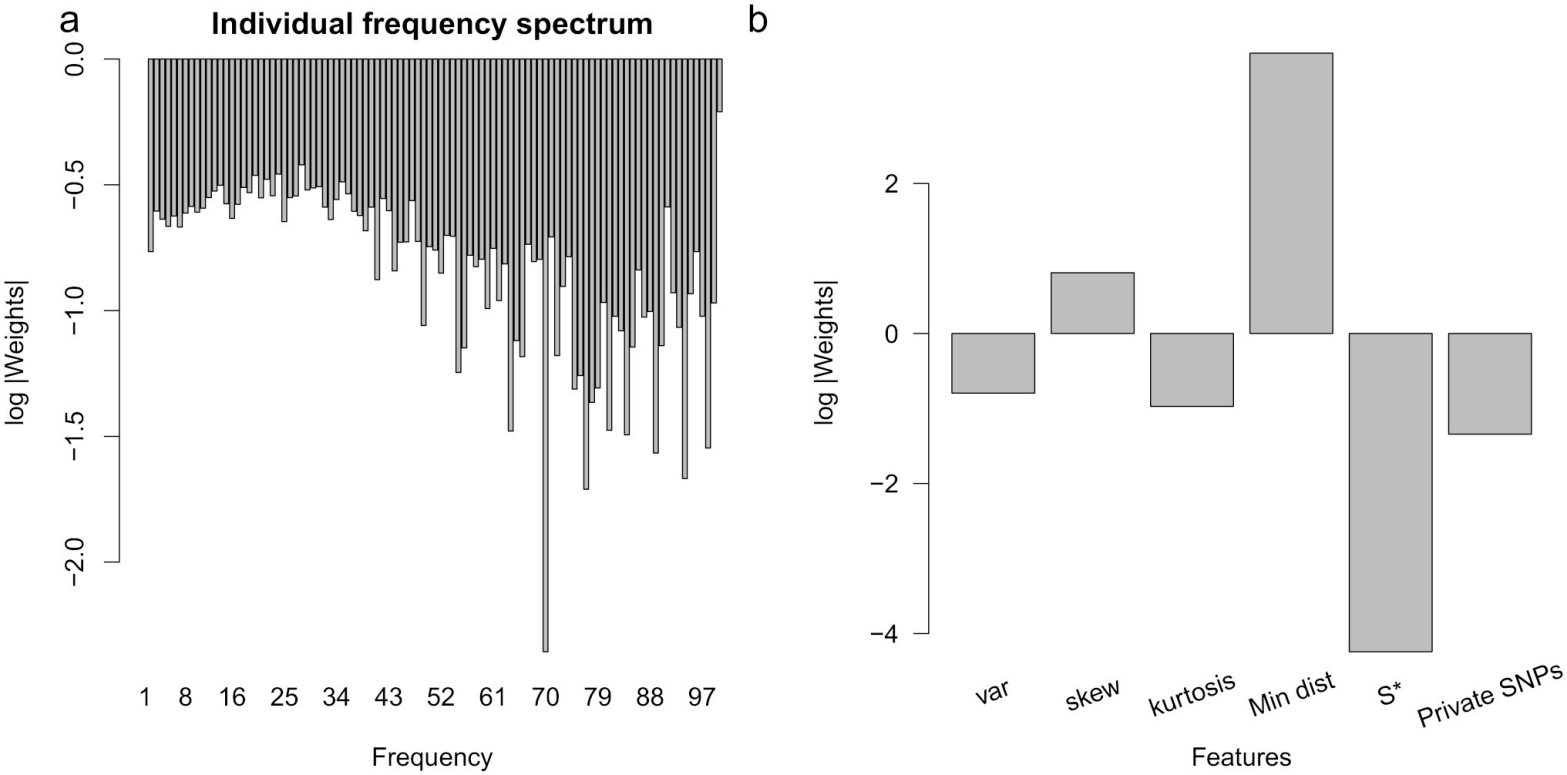
Relative importance of the features used as input to ArchIE. We examined the log of the absolute value of the standardized weights associated with each of the features included in the logistic regression model underlying ArchIE. Negative values indicate standardize weights with absolute values less than 1. (A) The individual frequency spectrum mostly has small weights and lower frequency entries generally have larger weights associated with them. (B) The first three entries indicate the moments of the distance vector. The minimum distance to the reference population, skew, and variance of the distance vector have the largest weights associated with them.

We examined the absolute value of the standardized weights learned by ArchIE to understand the features that contribute substantially to its predictions. Examining single features, we find that the minimum distance between the focal haplotype and each of the reference haplotypes, as well as the skew of the distance vector have the largest weights (Fig 3B). Intuitively, a larger distance to a reference population should indicate archaic ancestry. The next largest single statistic was the skew of the distance vector, which was negatively correlated with archaic ancestry. Under a simple scenario of admixture, we expect a bi-modal distribution of pairwise distances. However, when there is little archaic ancestry, the distribution will be unimodal resulting in a negative relationship between skew and archaic ancestry. The IFS contains mostly negative weights, suggesting that these features do not make a substantial contribution to the model predictions (Fig 3A).

As a further check, we wanted to determine how the performance of the model changes when trained on subsets of the features. First, since the “skew” feature has a large standardized absolute weight, we trained a model based only on this feature (S5 Fig). We find that accuracy greatly decreases, indicating that the model does best when it combines multiple features that are informative of archaic introgression. However, when we train only on the number of private SNPs or only on the minimum distance to the reference population, we see improved accuracy indicating that these features are informative of archaic ancestry independent of other features. When we take a combination of three features (skew, number of private SNPs, and minimum distance to the reference population), this model is still able to discern archaic from non-archaic haplotypes with slight decreased accuracy relative to the full model (S5 Fig). Finally, we tested the contribution of the reference population to the accuracy of ArchIE. We trained the logistic regression without using any features that rely on the reference and found that model still retains reasonable accuracy (AUPR = 0.36) to identify archaic ancestry (S5 Fig). This suggests that ArchIE is useful even in scenarios where a reference population is not available.

### Robustness of archaic local ancestry estimates

ArchIE relies on simulating data from a model with fixed demographic and population genetic parameters. In practice, these parameters are unknown and are inferred from data with some uncertainty. Thus, we wanted to determine the sensitivity of our method to demographic uncertainty. An exhaustive exploration of demographic uncertainty is challenging given the number of parameters associated with even the simplest models. As an alternative to an exhaustive exploration, we systematically perturbed each parameter at a time, simulated data using the perturbed model, and evaluated the performance of our classifier (trained on the unperturbed parameters corresponding to the Neanderthal demographic history).

**Fig 4:**
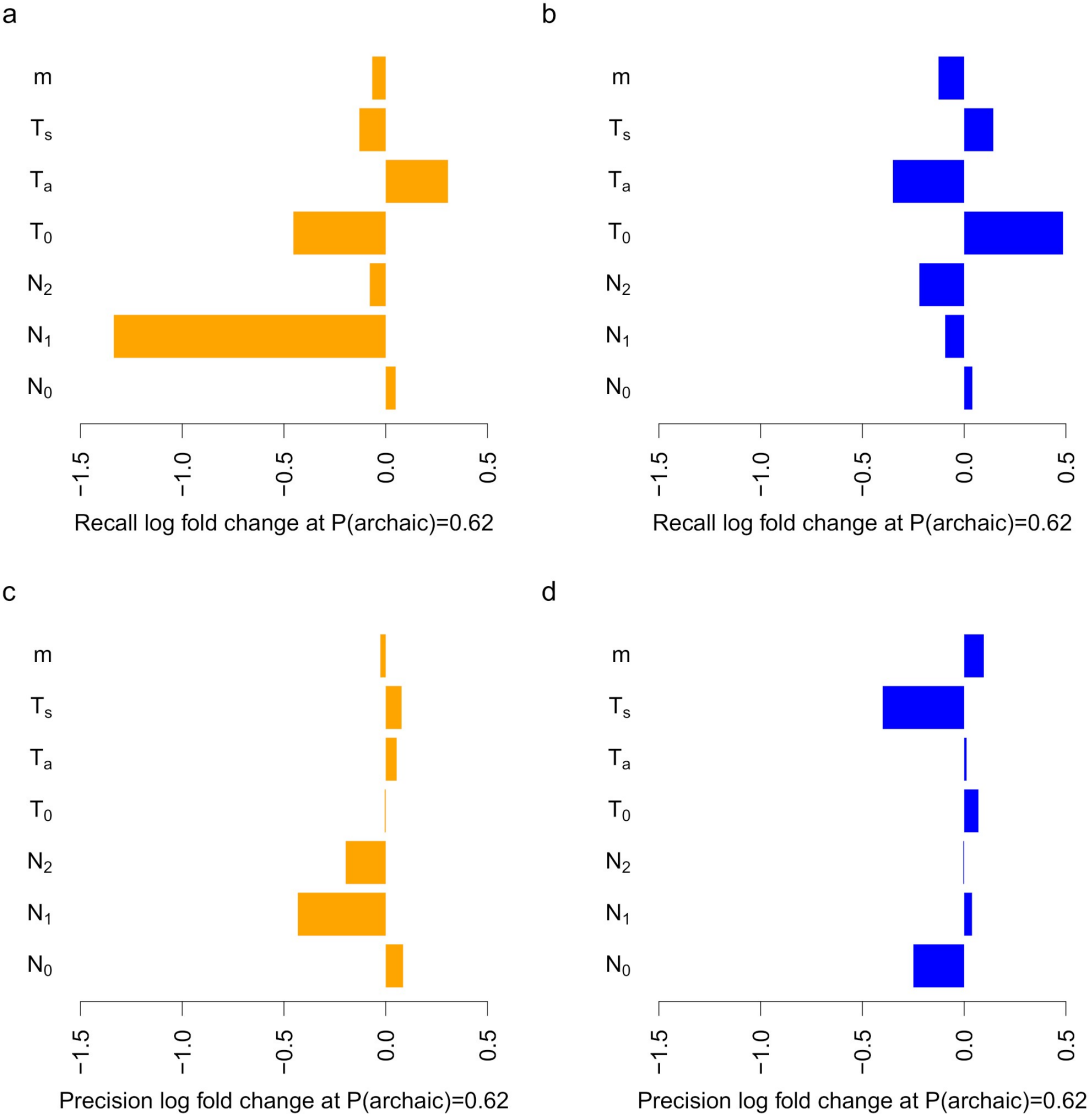
ArchIE is robust to misspecification in the demographic model. We tested ArchIE on data simulated after perturbing single demographic parameters lower (left, orange) and higher (right, blue) relative to their values in the training data. Values are reported as log_10_ fold changes compared to the baseline model performance. We report (a, b) recall and (c,d) precision at the threshold that gives a precision of 0.8 on the unperturbed test data (*P*(archaic) = 0.62).

ArchIE remains accurate when many aspects of the demography are misspecified, but has reduced precision or recall under some scenarios (Fig 4, S1 Fig). The most significant decrease in accuracy (in terms of recall and precision at a fixed threshold) arises when the reference population size is decreased or the split time of the reference and the target is increased. In this setting, the reference genomes are more drifted and hence, less representative of the ancestral population. We also compared the accuracies of ArchIE to S* across these perturbations and found that ArchIE remains relatively accurate across these settings (S1 Table).

We also tested the effect of variation in mutation rate (*μ*) and recombination rate (*r*) since we trained our model using fixed values of these parameters (*μ* = 1.25 × 10^−8^, *r* = 1 × 10^−8^). To evaluate how ArchIE performs on real data, we simulated test data randomly drawing pairs of *μ* and *r* from a distribution chosen to match local recombination and mutation rates along the human genome (see Methods). The overall AUPR is reduced (0.31, S1i Fig), the log_10_ fold changes in precision and recall are −0.30 and +0.19 suggesting that ArchIE is relatively robust to variation in mutation and recombination rates.

In addition, we tested the impact of the window size and found that reasonable choices of window size do not substantially impact the performance (S2 Fig). We also assessed the impact of sample size by simulating 30 haplotypes (15 diploid individuals), representing a modestly sized genomic dataset, and found a reduction in power as expected (AUPR = 0.45) (S3 Fig).

We tested the sensitivity of ArchIE to recent and ancestral structure in the demographic model. We simulated data under two scenarios of structure, one where 25% of the target population separates immediately after the target and reference population split, 2499 generations ago, and rejoins the generation prior to the archaic admixture, 2001 generations ago (S6A Fig). We refer to this as the recent structure scenario. Additionally, we simulated data where 25% of the population in *N*_0_ separates 12,000 generations ago and rejoins the ancestral population right before the target and reference populations split (2600 generations ago, S6B Fig). We refer to this as the ancestral structure scenario. We observe that for both scenarios, the fraction of SNPs detected as archaic is 0, suggesting that ArchIE is robust to introgression due to either recent or ancient structure at reasonable calling thresholds. We caution, however, that a more detailed exploration of structured demographic models is necessary.

### Reference-free detection of Neanderthal introgression in European populations

**Fig 5:**
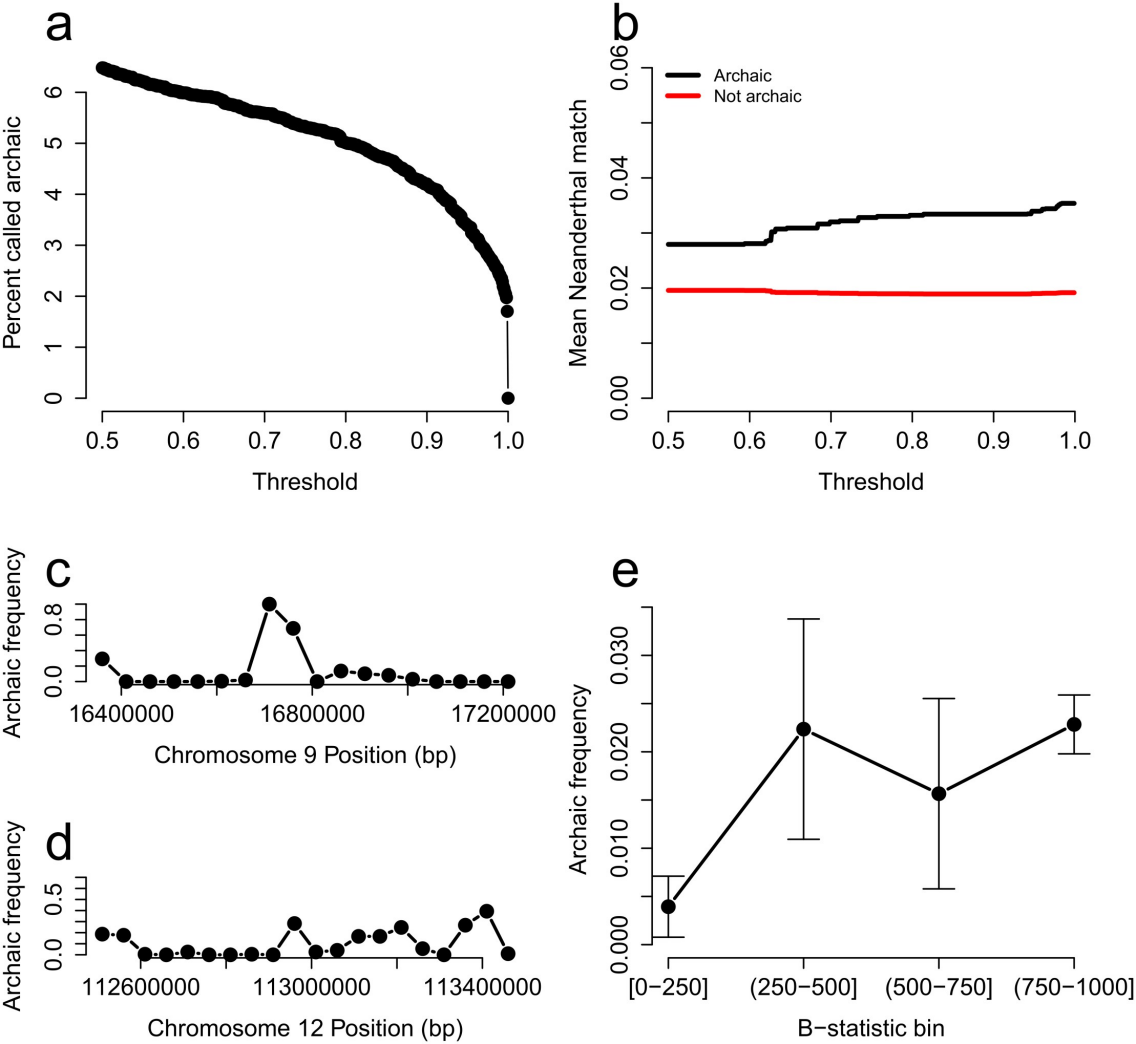
Application of ArchIE to 1000 Genomes European population (CEU). (A) Percentage of genome called archaic as a function of the threshold on the probability of archaic ancestry estimated by ArchIE. The dashed line refers to the threshold that yields a 20% FDR in simulations. (B) Mean Neanderthal match statistic (higher implies more similar to the sequenced Altai Neanderthal genome) for haplotypes inferred as archaic vs non-archaic as a function of the probability threshold. (C) Frequency of haplotypes confidently labeled as archaic near the *BNC2* gene and (D) the *OAS* gene cluster. (E) Mean frequency of confidently archaic segments increases with B-statistic (a measure of selective constraint). Low B-statistic denotes more selectively constrained regions (standard errors estimates are obtained using a 1 Mb block jackknife).

To identify segments of archaic ancestry in modern human populations, we applied ArchIE to genomes of European individuals in the 1000 Genomes Project [27]. We used all unrelated individuals from a European (CEU) population as our target population (99 diploid individuals) and all unrelated individuals from an African (YRI) population as a reference (108 diploid individuals) and calculated the summary statistics described above. We applied ArchIE in non-overlapping 50 Kb windows. We evaluated the average percent of windows inferred as archaic as a function of the calling threshold (Fig 5A). Applying a threshold corresponding to a precision of 0.80 in simulations, we inferred 2.04% (block jackknife SE = 0.6% using 1 Mb blocks) of the genome as confidently archaic. This proportion is in line with proportion of Neanderthal ancestry from previous analyses [2, 6, 10] suggesting that the segments of archaic ancestry inferred by ArchIE likely correspond to segments of Neanderthal ancestry.

To further investigate whether the haplotypes inferred as confidently archaic by our model are enriched for introgressed Neanderthal variants, we computed a Neanderthal match statistic (NMS) defined as the number of shared variants between an individual haplotype and the Altai Neanderthal reference genome sequence [10] divided by the total number of segregating sites in that window (see Methods). We see that the archaic regions confidently inferred by ArchIE have a higher NMS suggesting that the archaic ancestry segments identified by our method are likely to represent introgressed Neanderthal sequence (we reject the null hypothesis that the difference in NMS is zero for archaic vs non-archaic haplotypes with a *P* value = 1.7 × 10^−3^ via 100 Kb block jackknife). Further, as we make the calling threshold more strict, we see an increase in the mean NMS for the archaic haplotypes (Fig 5B).

We also compared the performance of ArchIE, S’, and S* on real data from CEU Europeans. For each of these methods, we computed a matching rate with the Altai Neanderthal genome, defined as the fraction of SNPs called archaic that match the Altai Neanderthal sequence divided by the total number of SNPs called archaic. At a detection rate of ≈ 1%, S’ has a matching rate of 0.73 while ArchIE has a matching rate of 0.91 (S9 Fig; see S1 Text for details). Comparing with the S* calls released from [28], we found a match rate of ≈ 50% at a detection rate of ≈ 0.5%, consistent with results reported from the authors.

We then focused on two genomic regions that have been shown to harbor introgressed Neanderthal haplotypes at elevated frequencies: the *BNC2* gene (Chromosome 9:16,409,501-16,870,786) [2] and the *OAS* gene cluster (Chromosome 12:113,344,739-113,357,712) [7]. ArchIE detects substantially increased frequency of archaic ancestry in both these genes (Fig 5C and 5D).

Finally, we analyzed the correlation between a measure of selective constraint of a given genomic region (B-value [23]) and frequency of confidently inferred archaic segments in the CEU population in the same region. Sankararaman *et al*. 2014 [2] describe a relationship where more constrained regions (lower B-value) have a lower frequency of archaic ancestry. We observe the same trend where more neutral regions (B-value ≥ 750) contain more archaic ancestry than constrained regions (B-value ≤ 250) consistent with selection against the archaic ancestry (*P* value = 7.86 × 10^−9^ via block jackknife; Fig 5E).

These analyses suggest that ArchIE obtains results concordant with those from a previous reference-aware method [2]. We caution, however, that the observed concordance can be inflated due to any biases shared by the two methods.

## Discussion

A key challenge in detecting the contribution of deeply-diverged populations (both deeply-diverged modern as well as archaic hominin populations) to the ancestry of present-day human populations arises from the lack of accurate representative genomes for these populations. Here, we present a statistical model (ArchIE) for detecting regions of archaic local ancestry without the need for an archaic reference sequence. ArchIE combines weakly informative signals computed from present-day human genomes using a logistic regression model. The parameters of the model are estimated from data simulated under a specific demographic model. Using simulations, we show that ArchIE obtains improved accuracy over other approaches for reference-free local ancestry inference. While the accuracy of ArchIE will depend on how similar the demographic model used for training is to the true demographic model, our empirical results suggest that ArchIE is relatively robust even when the true demographic model differs from the assumed model. Applying ArchIE to genomes from the CEU population in the 1000 Genomes project data, we detect 2.03 ± 0.6% archaic ancestry (at a threshold that corresponds to a false discovery rate of 0.2). We find that segments confidently labeled as archaic by ArchIE are enriched for Neanderthal ancestry.

One advantage of our approach is that the learning algorithm is general allowing it to be applied broadly to diverse inference problems as well as input summary statistics while its simplicity allows for a transparent interpretation of the features and the model.

There are several limitations of our methodology, however. First, we require some knowledge of the demographic history of the target, reference and archaic populations. We have shown that ArchIE is robust to some demographic misspecification, but it is most powerful when the simulated demography is close to the true one. Second, we rely on the data being phased. Switch-errors in phasing will reduce the power of ArchIE, which can be a problem when applying the method to less-well studied populations. In principle it is possible to use ArchIE on unphased data, calculating features on the diploid individual level rather than the haplotype level, though we do not explore that here. Third, the use of a fixed-size window ignores long-range as well as variable-length dependence among the features. Models that account for this dependency can be expected to yield improved accuracy. An example of such an approach is a recently published method that uses a hidden Markov model (HMM) that models the distribution of private variants [12]. Combining such models with the framework outlined here has the potential to yield improved accuracies. Fourth, the use of a linear model is likely to underfit the true function between features and outputs. It is possible to train more expressive models like deep neural networks, which can learn and capture non-linear relationships between features and tend not to suffer from the curse of dimensionality [19]. These methods have been used to great success in tasks such as image classification [29] and we anticipate their use in population genetics could improve predictive power. Preliminary results applying deep learning to this problem with the features used here are promising, motivating future work (S1 Text, S7 and S8 Figs). ArchIE relies on a careful choice of features as input. These hand crafted features are informed by population genetics theory, similar to other methods that have been proposed in population genetics [19, 20, 30, 31, 14]. Automatically learning features from genetic data is direction of high interest. Finally, while several methods [9, 12, 22] have been proposed to infer aspects of archaic ancestry without access to reference genomes, these methods are typically evaluated using simulations. Assessing the accuracy of these methods on real data remains challenging. Extrapolating simulation results to accuracy on real data depends on choices of the inference problem, population genetic models, parameters used for training and testing, genomic features used as input, and accuracy metrics of interest. A comprehensive comparison of these methods across a range of demographic histories and evolutionary forces is an important topic for future work.

In conclusion, our method improves on previous methods for reference-free inference of archaic ancestry by combining informative summary statistics in a statistical learning framework. We anticipate that this method will be informative not only in human populations where questions about admixture with other hominins abound, but also in other species and systems where pervasive admixture has shaped the distribution of genetic variation.

## Methods

### Simulating training data

We simulated training and test data sets using a modified version of ms [24] that tracks the ancestry of each site in each individual genome. Using a previously proposed demographic model relating modern humans and Neanderthals [2], we sampled 100 haplotypes from the target, and 100 haplotypes from the reference over a region of length 50 Kb. We use a constant mutation rate *μ* = 1.25 × 10^−8^ and a recombination rate *r* = 1 × 10^−8^.

The general demography is as follows: an archaic population of size *N*_*a*_ splits from a population of size *N*_0_, *T*_0_ generations before present (B.P.). Then, at *T*_*S*_, two populations split off from the ancestral population that then have effective population sizes *N*_1_ (termed the reference) and *N*_2_ (termed the target) respectively. Then, at time *T*_*A*_, the archaic population migrates into the target with an admixture fraction *m*. See Fig 1 for a graphical outline.

### Feature calculation

Each simulation at a given locus generates 100 haplotypes in the target. For each haplotype, we calculate the following classes of summary statistics: individual frequency spectrum, distance vector to all haplotypes within the test population as well as the first four moments of this vector, minimum distance to haplotypes in the reference population, the number of private SNPs, and the S*-statistic.

The individual frequency spectrum is created as follows: given a sample of *n* haplotypes, for each haplotype *j*, we construct a vector *X* of length *n* where entry *X*_*i*_ counts the number of derived alleles carried on the focal haplotype *j* whose derived allele frequency is *i*. For example, the first entry counts the number of singletons present in haplotype *j*, the second entry counts the number of doubletons and so on until *n*.

The distance vector is a vector of length *n* where entry *i* is the Euclidean distance from haplotype *j* to haplotype *i* over all sites, where *j* is the focal haplotype and *i* is the haplotype being compared.

The minimum distance to haplotypes in the reference population is computed as the minimum Euclidean distance from the focal haplotype to all haplotypes in the reference population.

The number of private SNPs is calculated as the number of SNPs the focal haplotype contains that are not present in the reference population.

This results in 208 features per example (a 50 Kb window for a single haploid genome), with 100 examples per locus and 10,000 loci resulting in 1,000,000 examples for training before filtering haplotypes with intermediate levels of admixture.

### Learning algorithm

We used the “glm” function in R to construct a logistic regression model using the family = binomial(“logit”) option. We used the predict function to obtain a prediction and converted it to a probability using the “plogis” function.

Due to the process of recombination, the ancestry of a haplotype may vary along its length. On the other hand, ArchIE predicts a single ancestry state for a haplotype across a specified window. We evaluate the ability of ArchIE to predict the ancestry at each SNP along a haplotype by simulating sequences of length 1 Mb and applying ArchIE in 50 Kb windows, sliding by 10 Kb at a time. We average the predictions that each SNP on a haplotype receives across all windows that overlap the SNP to obtain the predicted archaic ancestry. We compare the predicted and the true ancestry state at each SNP along a haplotype.

We evaluated the performance using Precision-Recall (PR) curves as well as receiver operator characteristic (ROC) curves. We calculated precision (equivalently 1− the false discovery rate), recall (equivalently sensitivity) and false positive rates as:

$$\begin{array}{c} Recall\left(t\right)=\frac{TP(t)}{TP(t)+FN(t)} \end{array}$$

$$\begin{array}{c} Sensitivity\left(t\right)=Precision\left(t\right)=\frac{TP(t)}{TP(t)+FP(t)} \end{array}$$

$$\begin{array}{c} False\;positive\;rate\left(t\right)=\frac{FP(t)}{FP(t)+TN(t)} \end{array}$$

Here *TP*(*t*) is the number of true positives at threshold *t*, *FN*(*t*) is the number of false negatives at threshold *t*, *FP*(*t*) is the number of false positives at threshold *t* and *TN*(*t*) is the number of true negatives at threshold *t*. We summarize these results by reporting the recall at a fixed value of precision as well as by computing the area under the precision recall curve (AUPR) and the area under the ROC curve (AUROC). We compute the AUPR using the method of Davis and Goadrich [32]. We compute standard errors of the AUPR and AUROC using a block jackknife [26] where we drop a single 1 Mb region and recompute the statistics.

### Comparisons

We compared ArchIE to the S* [9] and S’ [22] statistics. We calculate S* in a cohort of 100 haplotypes from the target population. Then, we convert the S* scores into a rank between [0-1] using the empirical cumulative distribution. We use a 50 Kb sliding window (10 Kb stride) across the 1 Mb region, averaging the score for a SNP.

We use a similar strategy for S’. However, since S’ predicts archaic ancestry in a sample of individuals rather than on the haploid genome level, we use an algorithm to convert sample predictions to haploid genome predictions. We run S’ on the sample. Then, at some S’ score threshold, we find the longest stretch of SNPs at that score or higher and interpolate the scores across genotypes, building haplotypes when individuals have the archaic allele. Then, for each SNP, we evaluate whether the SNP is archaic or not and calculate the number of true positives, false positive, true negatives, and false negatives. We repeat this procedure across thresholds and calculate the precision, recall, and false positive rates.

### Robustness

We examined the robustness of ArchIE to a specified demographic model by systematically perturbing one parameter at a time, simulating a dataset, and evaluating ArchIE’s performance. We doubled and halved the parameters, except when doing so would produce a demographic model that is not sensible.

We evaluated the robustness of ArchIE to mutation and recombination rate variation by calculating local rates at 50 Kb windows and then randomly drawing combinations of the rates and simulating data. Mutation rates were calculated by estimating Watterson’s *θ* [33] from the number of segregating sites within 50 Kb windows across 50 randomly sampled west African Yoruba genomes from the 1000 Genomes Project Phase 3 release and calculating the mutation rate: *μ* = *θ*_*w*_/4*N*_*e*_*L* where we set *N*_*e*_ = 10, 000. Recombination rates were estimated from the combined, sex-averaged HapMap recombination map [34].

### Neanderthal introgression

We validated our method using the Neanderthal introgression scenario as a test case. We downloaded phased CEU genomes from the 1000 Genomes Phase 3 dataset [27] and calculated the features mentioned above in 50 Kb windows. For each individual haplotype, we inferred the probability that the window is archaic. We then intersected our calls with the 1000 Genomes strict mask using BEDtools v2.26.0 [35], removing regions that are difficult to map to, measured as having less than 90% of sites in the callability mask.

We calculated a Neanderthal match statistic (NMS) for focal haplotype *i* in a window as the fraction of alleles at which the the focal haplotype matches the Altai Neanderthal [10] genome:

$$\begin{array}{c} NMS_{i}=\frac{S_{i}}{N_{i}+H_{i}} \end{array}$$

Here *S*_*i*_ denotes the number of alleles that match between the focal haplotype and the Neanderthal genome within the window. Since the Neanderthal genome is not phased, we count sites as matching if it contained at least one single matching allele or more. *N*_*i*_ denotes the number of Neanderthal mutations, including both homozygous and heterozygous sites. *H*_*i*_ denotes the number of human mutations within the window.

In order to test whether there is more Neanderthal matching in archaic haplotypes compared to non-archaic haplotypes, we computed the difference in NMS between the two classes of haplotypes at each window and test the hypothesis that the mean of this statistic averaged across the genome is zero. Specifically:

$$\begin{array}{c} \Delta_{NMS,i}=\frac{{\bar{NMS}}_{arch,i}-{\bar{NMS}}_{non-arch,i}}{\bar{NMS_{i}}} \end{array}$$

For each window *i*, we compute Δ_*NMS*,*i*_, defined as the difference between the mean NMS for archaic (${\bar{NMS}}_{arch,i}$) and non-archaic (${\bar{NMS}}_{non-arch,i}$) haplotypes divided by the mean NMS of all haplotypes ($\bar{NMS_{i}}$) to control for mutation rate heterogeneity. We require a minimum of 90% callable sites within the window. We compute the mean of Δ_*NMS*,*i*_ over all windows *i* as the genome-wide estimate and test if this estimate is significantly different from zero. To compute significance, we use a block jackknife and drop non-overlapping 100 Kb windows and recalculate the genome wide difference in means.

### Background selection

In order to assess the relationship between background selection and inferred archaic ancestry, we use the B-values from McVicker *et al*. 2009 [23] and intersected them with our calls. For visualization, we binned the B-values into 4 bins, [0-250], (250-500], (500-750], and (750-1000].

We tested for significant differences in allele frequency between the lowest and highest bins using a block jackknife using a 50 Kb block size.

## Supporting information

S1 Fig Precision-Recall curves when the distribution of the test data differs from the training data used for estimating the parameters of ArchIE. We perturbed a single parameter associated with the simulations used for generating training data. *m* is the admixture fraction from the archaic into the target population. *N*_0_ is the ancestral population size. *N*_1_ is the size of the reference population and *N*_2_ is the size of the target population. *T*_0_ refers to the split time of the archaic and modern human population. *T*_*s*_ is the split time of the reference and target populations. *T*_*a*_ is the admixture time and mu rho refers to the experiment that uses realistic recombination and mutation rates, estimated from the human genome (see Methods for more details).
(PDF)

S2 Fig Robustness to changing window size. ArchIE obtained similar accuracies when applied with window sizes of 100 Kb and 25 Kb relative to the 50 Kb case (‘Unperturbed’).
(PDF)

S3 Fig Robustness to smaller sample sizes. We evaluated how ArchIE performs with 30 haplotypes (15 diploid individuals). We see that ArchIE loses power when the sample size is greatly reduced.
(PDF)

S4 Fig Precision-Recall and Receiver Operating Characteristic curves for haplotype-level predictions. We evaluated ArchIE’s ability to predict entire haplotypes as archaic (as opposed to archaic ancestry at each SNP in Fig 2). A haplotype is labeled as truly archaic if ≥ 70% of its bases are archaic in ancestry and not archaic if ≤ 30 is labeled archaic. We ignore haplotypes with intermediate values of archaic ancestry from our comparisons. We used haplotypes of length 50 Kb.
(PDF)

S5 Fig Precision-Recall curves for different sets of input features. In ‘No MH Ref’, we removed the features that rely on the reference population. The resulting predictor has reasonable albeit reduced accuracy relative to ArchIE (labeled “Full”). We evaluated the predictive accuracy of a logistic regression model trained with only a single feature where we considered the skew feature (“skew only”), the private SNPs feature (“P SNPs only”), and the minimum distance to the reference (”Min. D only”). Accuracy is substantially decreased for “skew only” while using only the private SNPs feature (‘P SNPs only’) or the minimum distance to the reference (‘Min. D only’) results in good performance, especially at the high precision regime. In ‘3 feat.’, we use skew, minimum distance, and private SNPs as the only features. While this set achieves good performance, adding the full set of features still outperforms this set of three features. Area under the PR curve (AUPR) is shown in parenthesis.
(PDF)

S6 Fig Demographic models for (A) recent structure and (B) ancient structure. (PDF)

S7 Fig Neural network architecture and training procedure. (PDF)

S8 Fig Neural network performance. Precision-recall curves for a 2% admixture scenario. Performance of the neural network is shown in blue.
(PDF)

S9 Fig Comparison of ArchIE, S’, and S* in 1000G CEU individuals. (PDF)

S1 Table Robustness to demographic misspecification. We simulated data under misspecified demographies, perturbing each parameter separately and evaluated the performance of S* and ArchIE. We present precision and recall at a threshold that corresponds to a precision of 0.8 (20% FDR) in the unperturbed setting. Bold denotes settings where ArchIE is higher precision as well as recall over S*.
(XLSX)

S1 Text Neural network model description and comparison of ArchIE with S’ and S* in 1000G data. (PDF)
