# Supplementary figures and images for: A statistical model for reference-free inference of archaic local ancestry
Source: PLoS Genet. 2019 May 28;15(5):e1008175. doi: 10.1371/journal.pgen.1008175 (PMC6555542; doi:10.1371/journal.pgen.1008175)

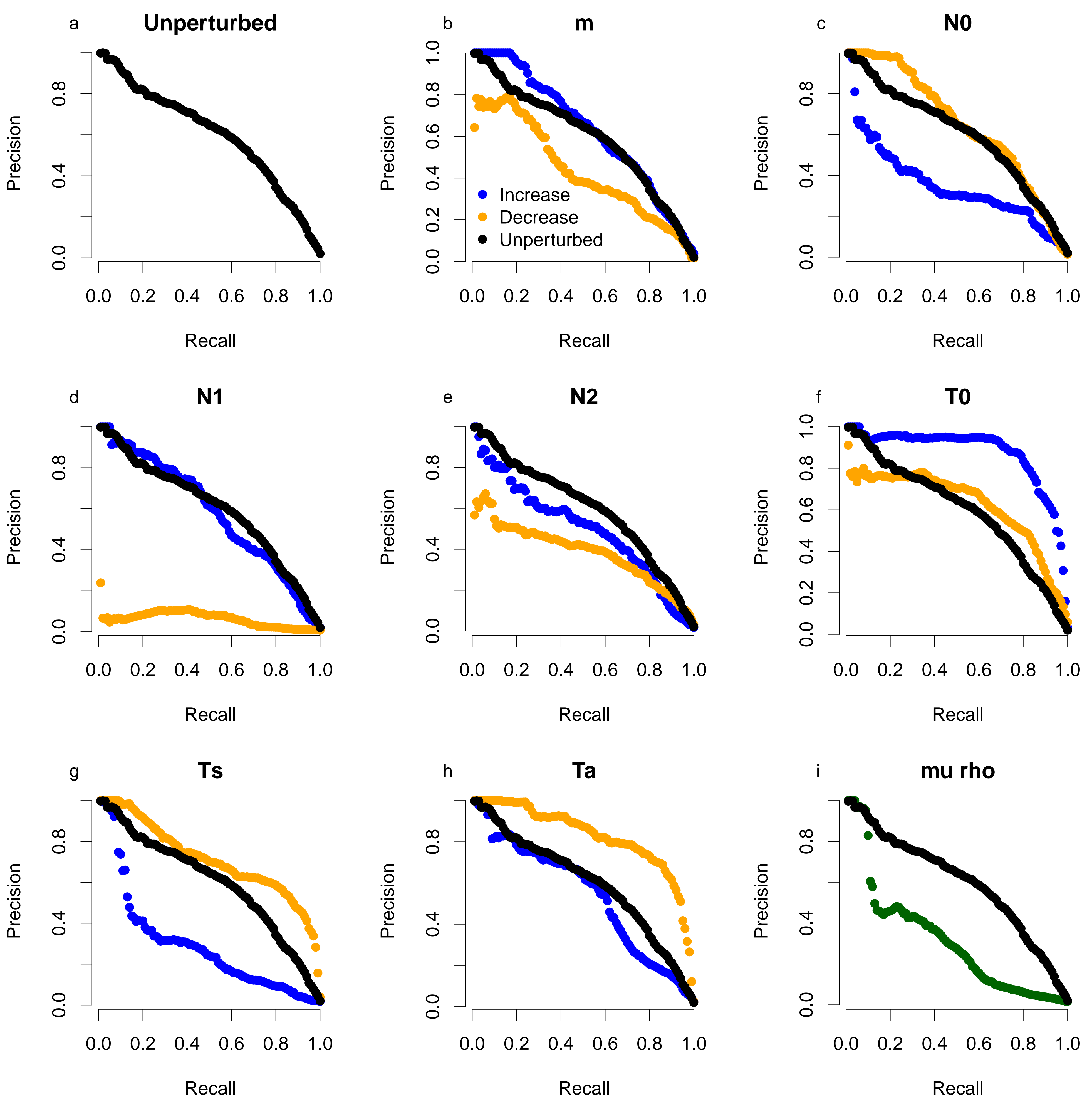

Supplement: S1 Fig — We perturbed a single parameter associated with the simulations used for generating training data. m is the admixture fraction from the archaic into the target population. N0 is the ancestral population size. N1 is the size of the reference population and N2 is the size of the target population. T0 refers to the split time of the archaic and modern human population. Ts is the split time of the reference and target populations. Ta is the admixture time and mu rho refers to the experiment that uses realistic recombination and mutation rates, estimated from the human genome (see Methods for more details). (PDF) [file pgen.1008175.s001.pdf]

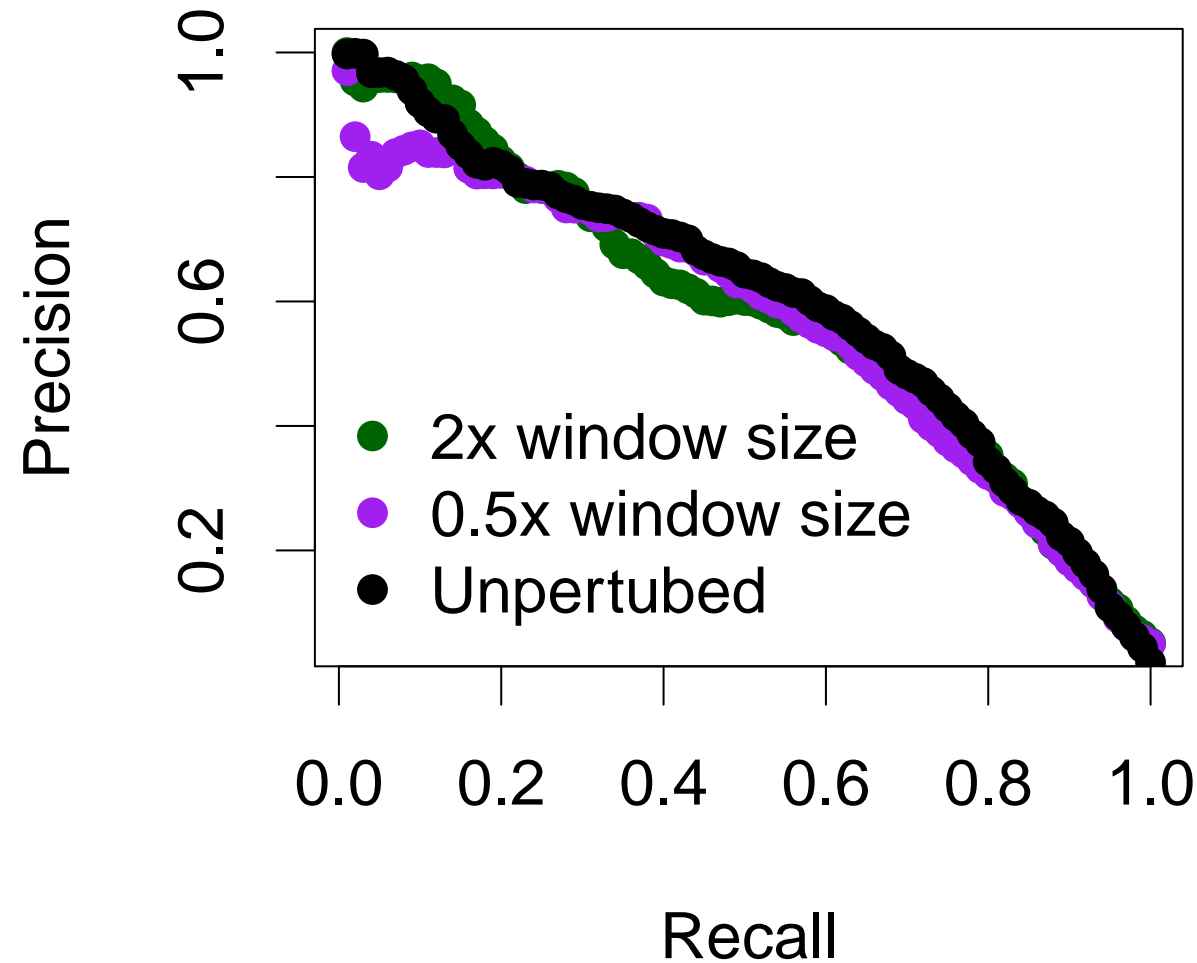

Supplement: S2 Fig — ArchIE obtained similar accuracies when applied with window sizes of 100 Kb and 25 Kb relative to the 50 Kb case (‘Unperturbed’). (PDF) [file pgen.1008175.s002.pdf]

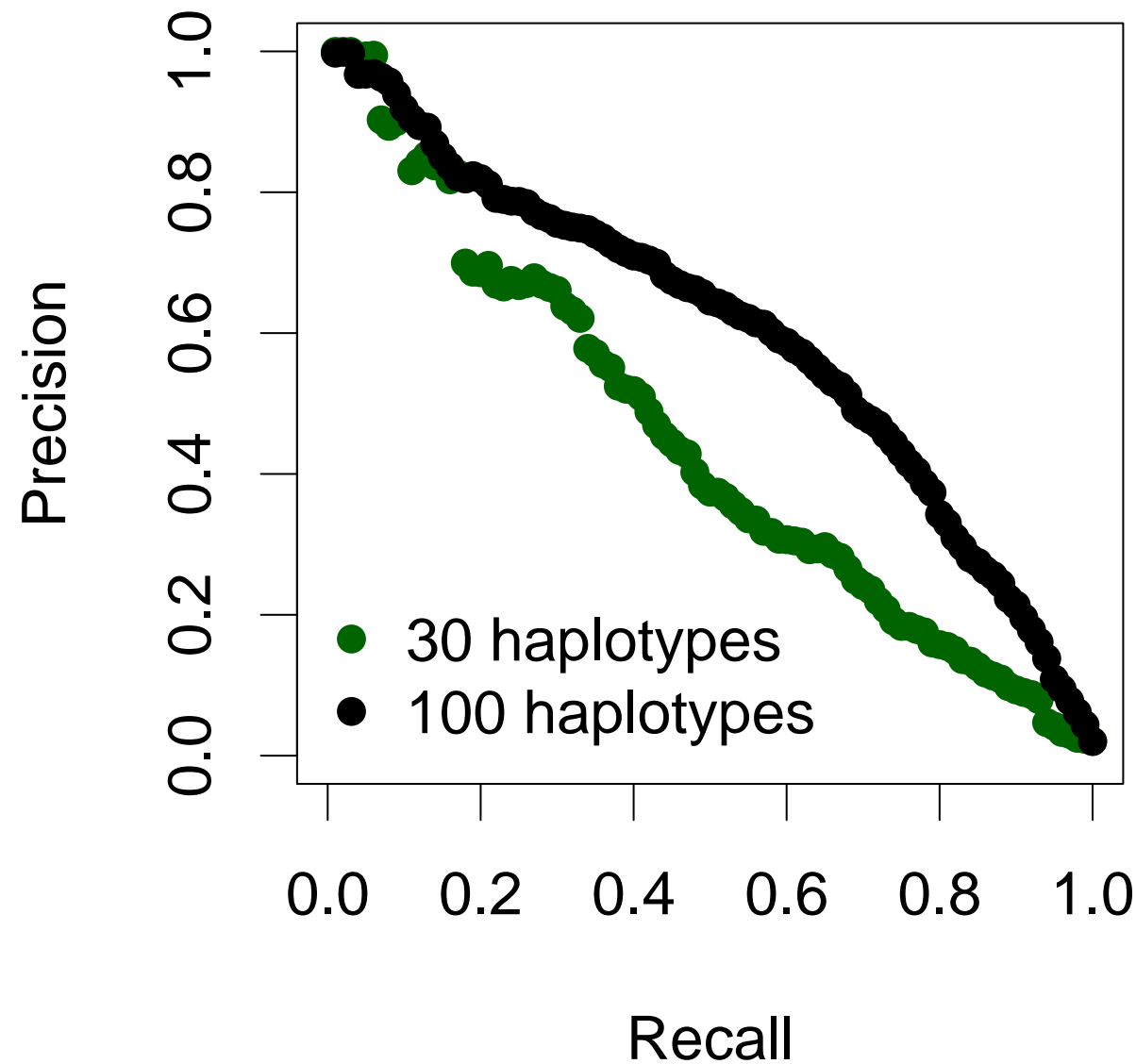

Supplement: S3 Fig — We evaluated how ArchIE performs with 30 haplotypes (15 diploid individuals). We see that ArchIE loses power when the sample size is greatly reduced. (PDF) [file pgen.1008175.s003.pdf]

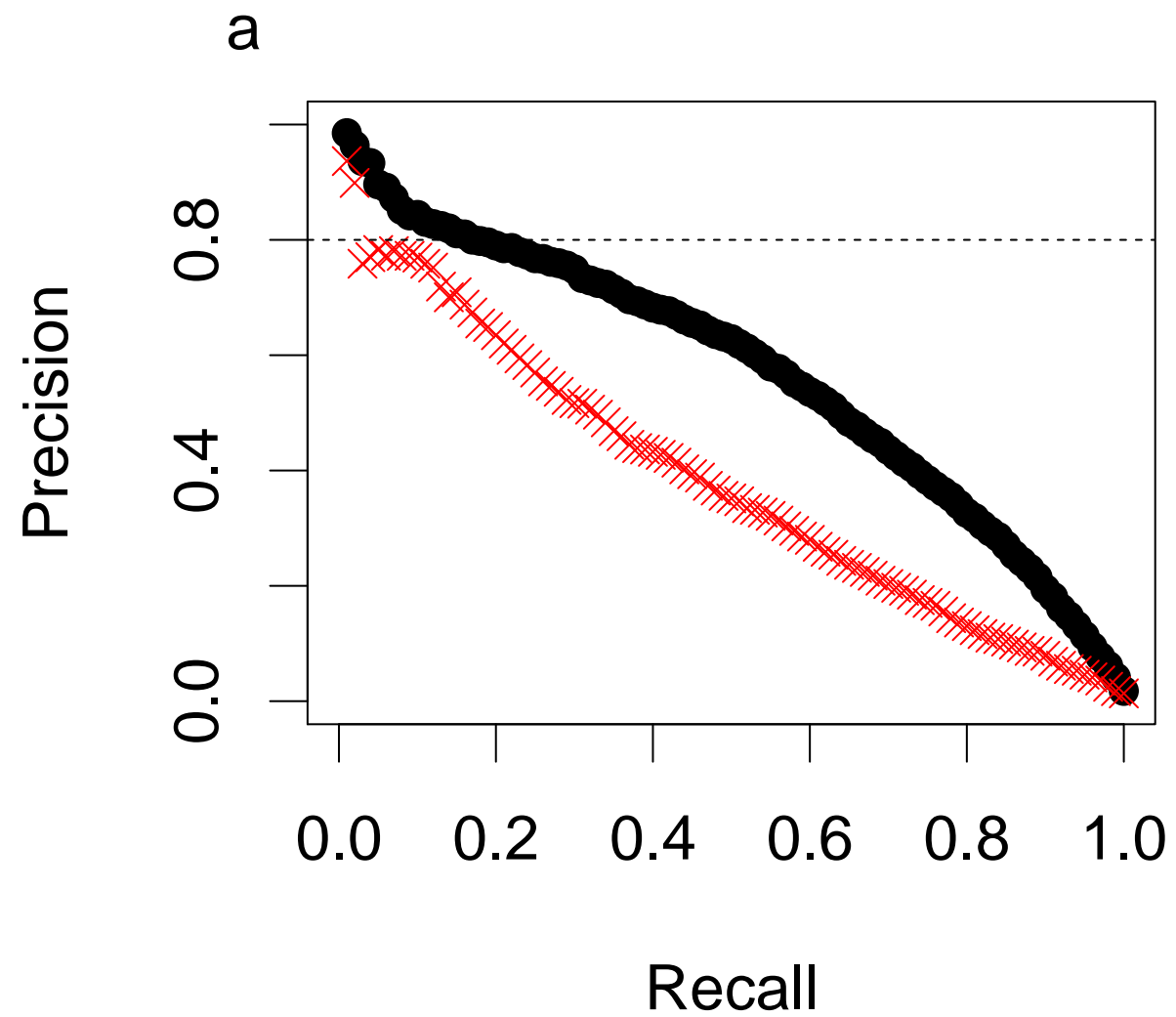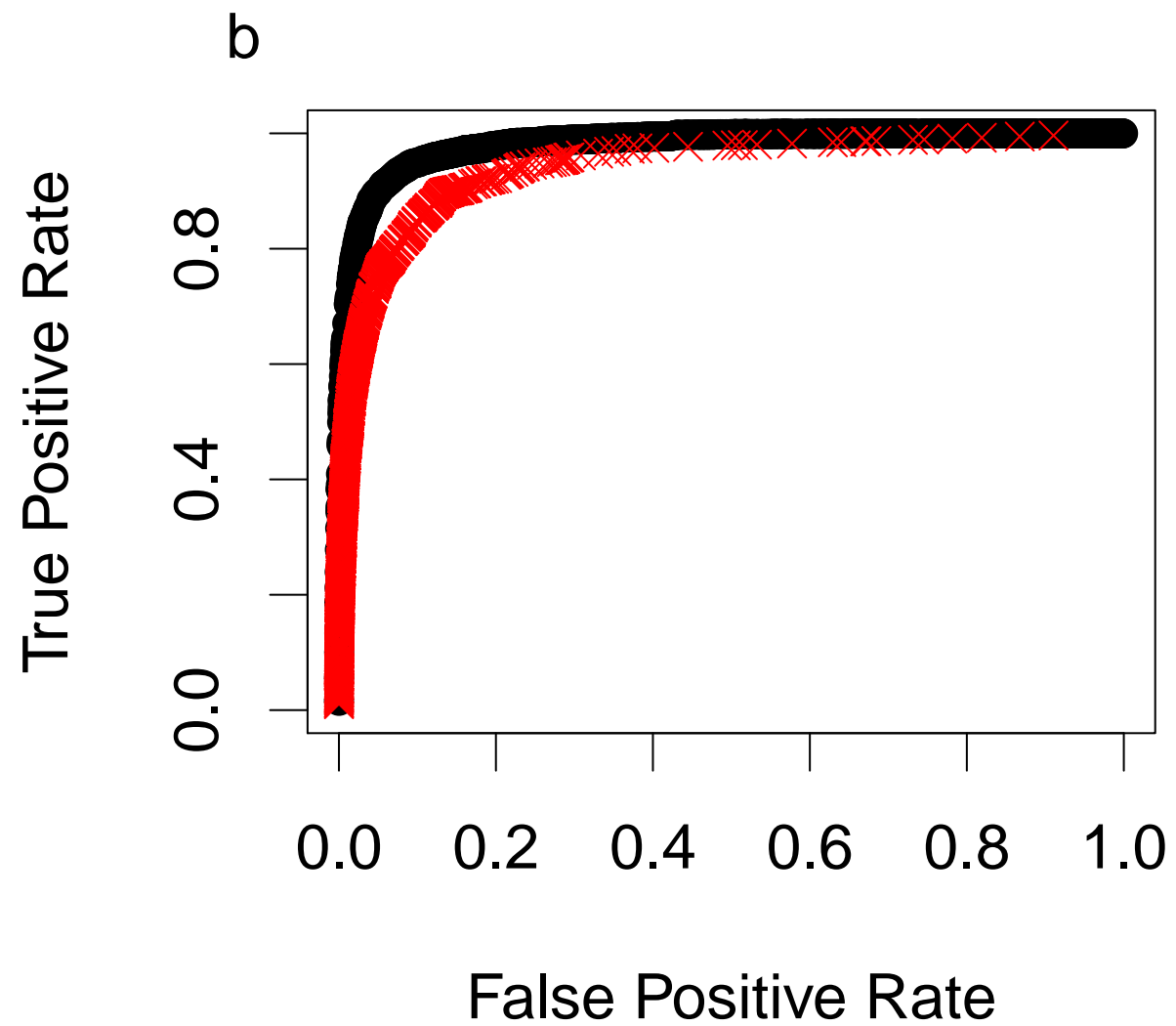

Supplement: S4 Fig — We evaluated ArchIE’s ability to predict entire haplotypes as archaic (as opposed to archaic ancestry at each SNP in Fig 2). A haplotype is labeled as truly archaic if ≥ 70% of its bases are archaic in ancestry and not archaic if ≤ 30 is labeled archaic. We ignore haplotypes with intermediate values of archaic ancestry from our comparisons. We used haplotypes of length 50 Kb. (PDF) [file pgen.1008175.s004.pdf]

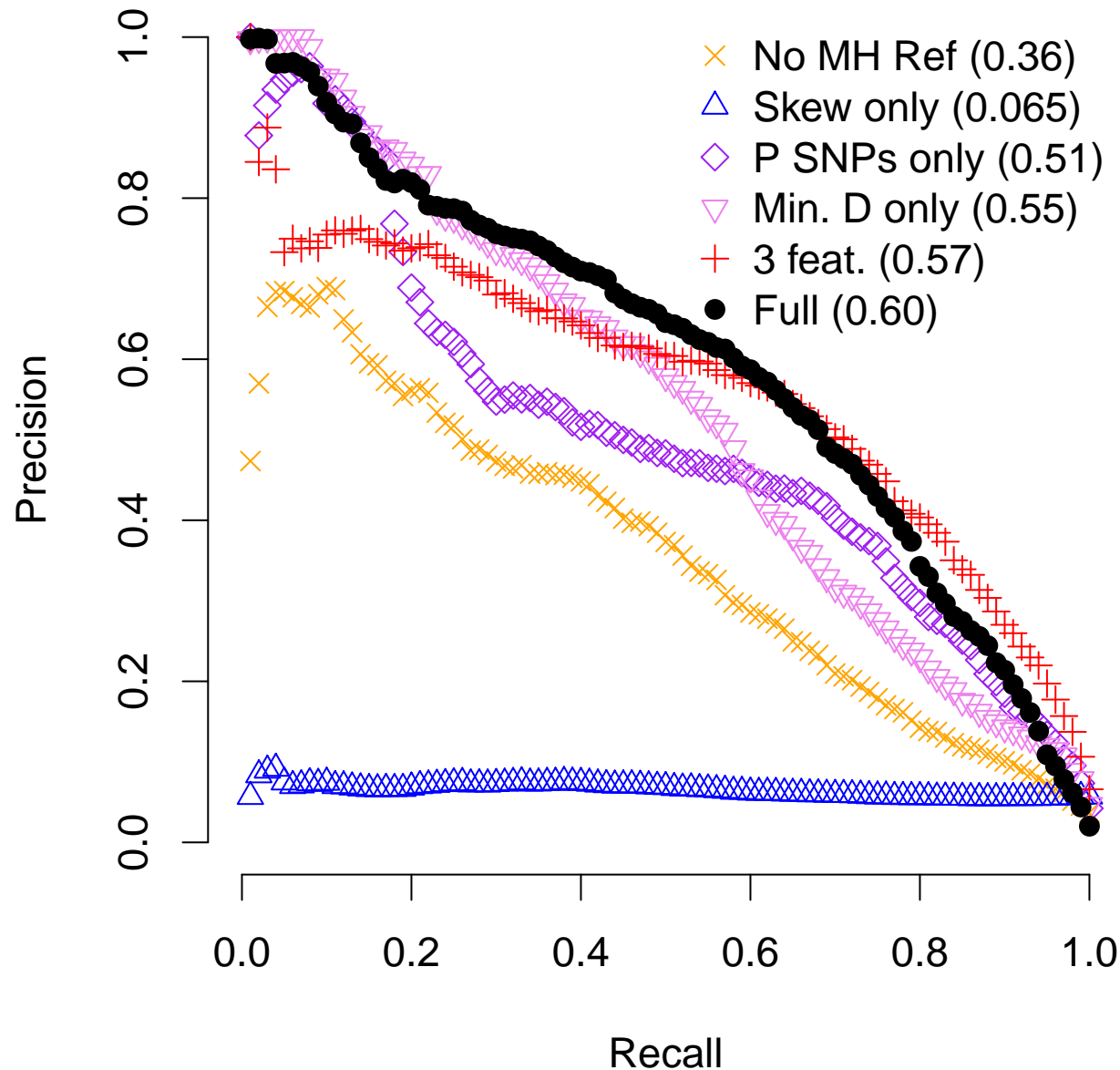

Supplement: S5 Fig — In ‘No MH Ref’, we removed the features that rely on the reference population. The resulting predictor has reasonable albeit reduced accuracy relative to ArchIE (labeled “Full”). We evaluated the predictive accuracy of a logistic regression model trained with only a single feature where we considered the skew feature (“skew only”), the private SNPs feature (“P SNPs only”), and the minimum distance to the reference (”Min. D only”). Accuracy is substantially decreased for “skew only” while using only the private SNPs feature (‘P SNPs only’) or the minimum distance to the reference (‘Min. D only’) results in good performance, especially at the high precision regime. In ‘3 feat.’, we use skew, minimum distance, and private SNPs as the only features. While this set achieves good performance, adding the full set of features still outperforms this set of three features. Area under the PR curve (AUPR) is shown in parenthesis. (PDF) [file pgen.1008175.s005.pdf]

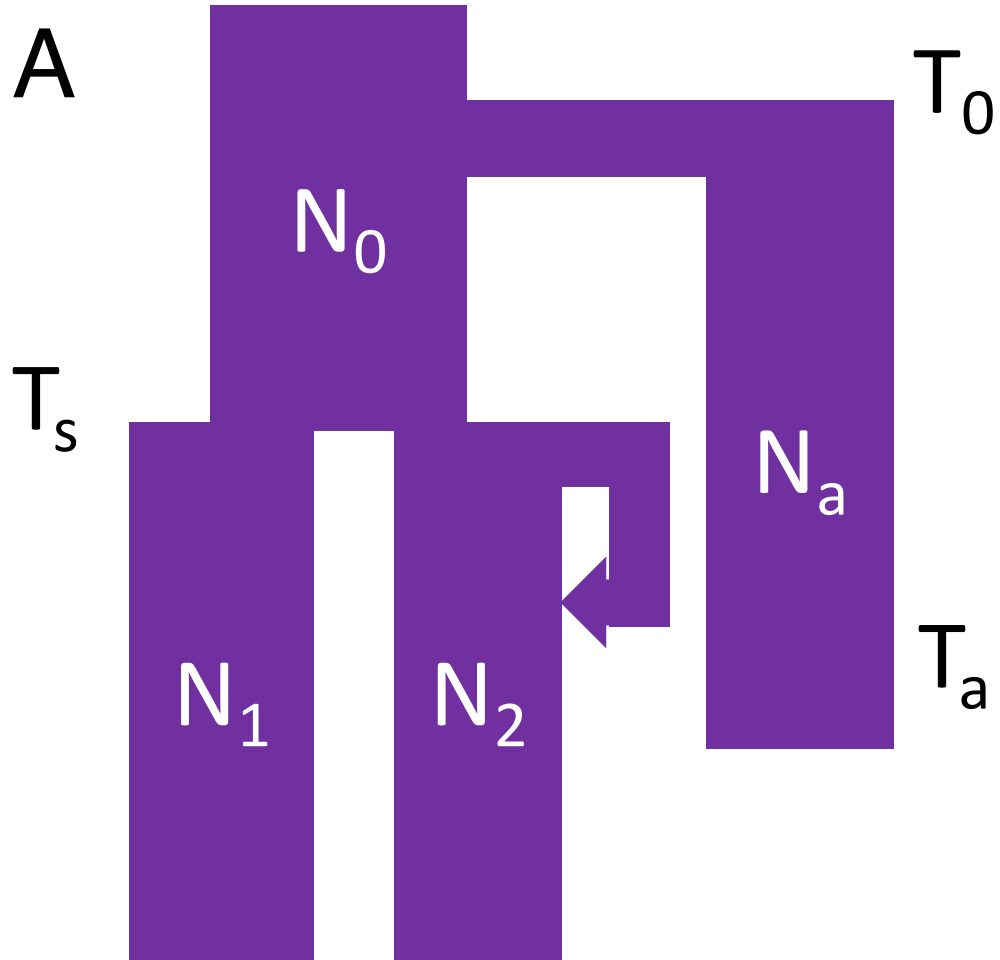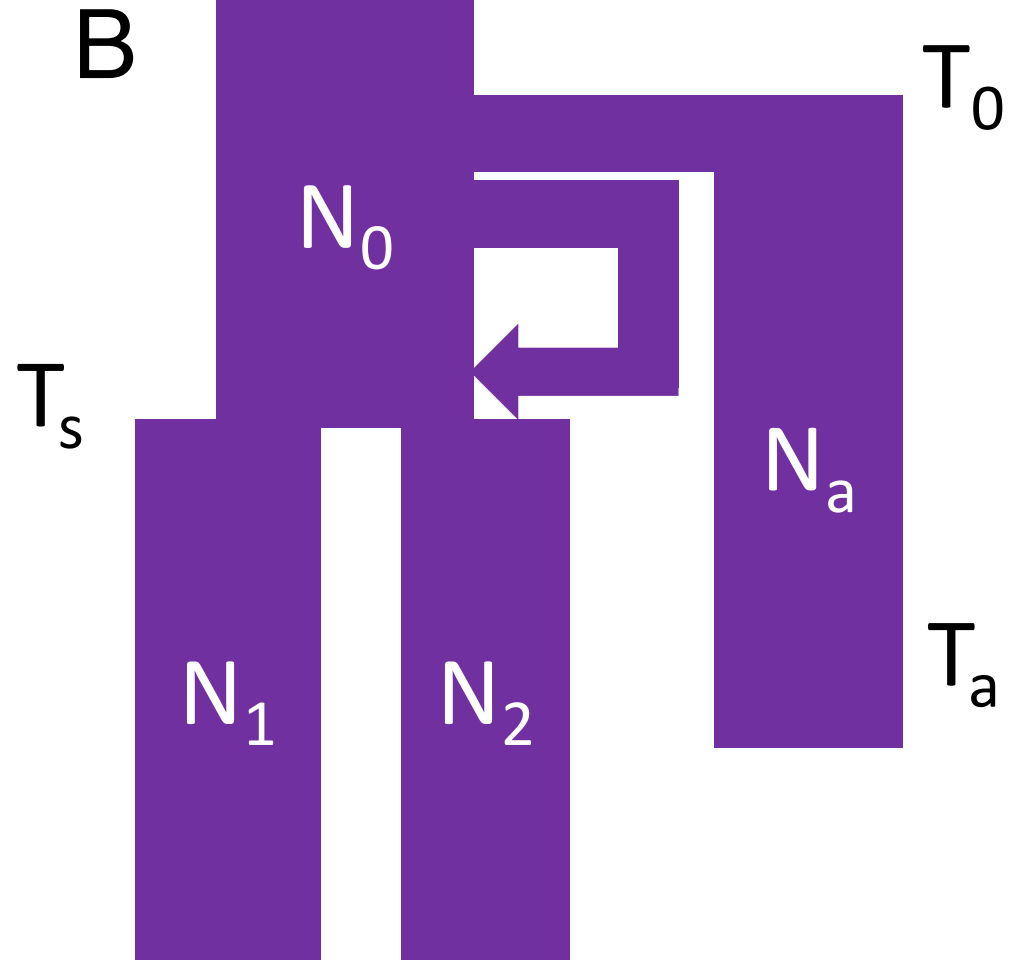

Supplement: S6 Fig — (PDF) [file pgen.1008175.s006.pdf]

## A. Coalescent simulations

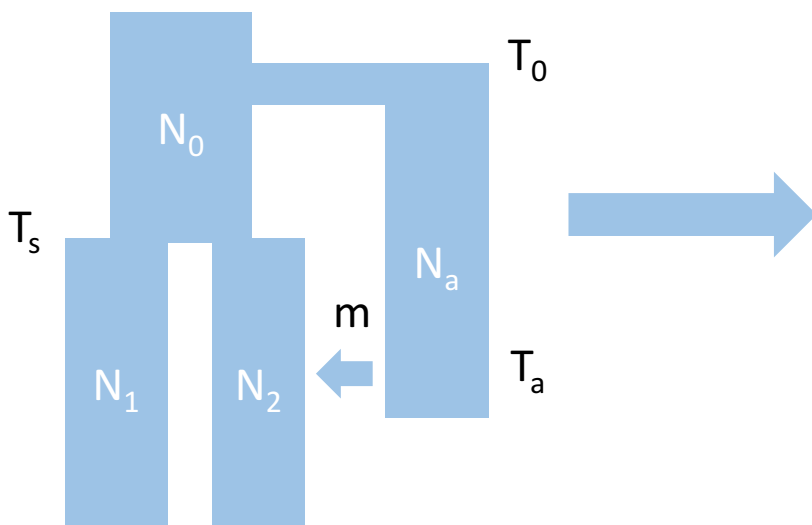

## B. Feature calculation

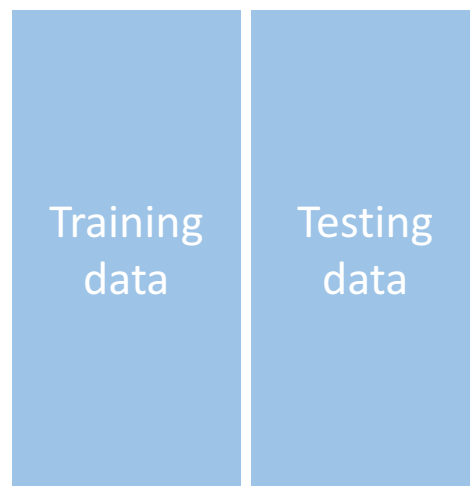

## C. Neural network training

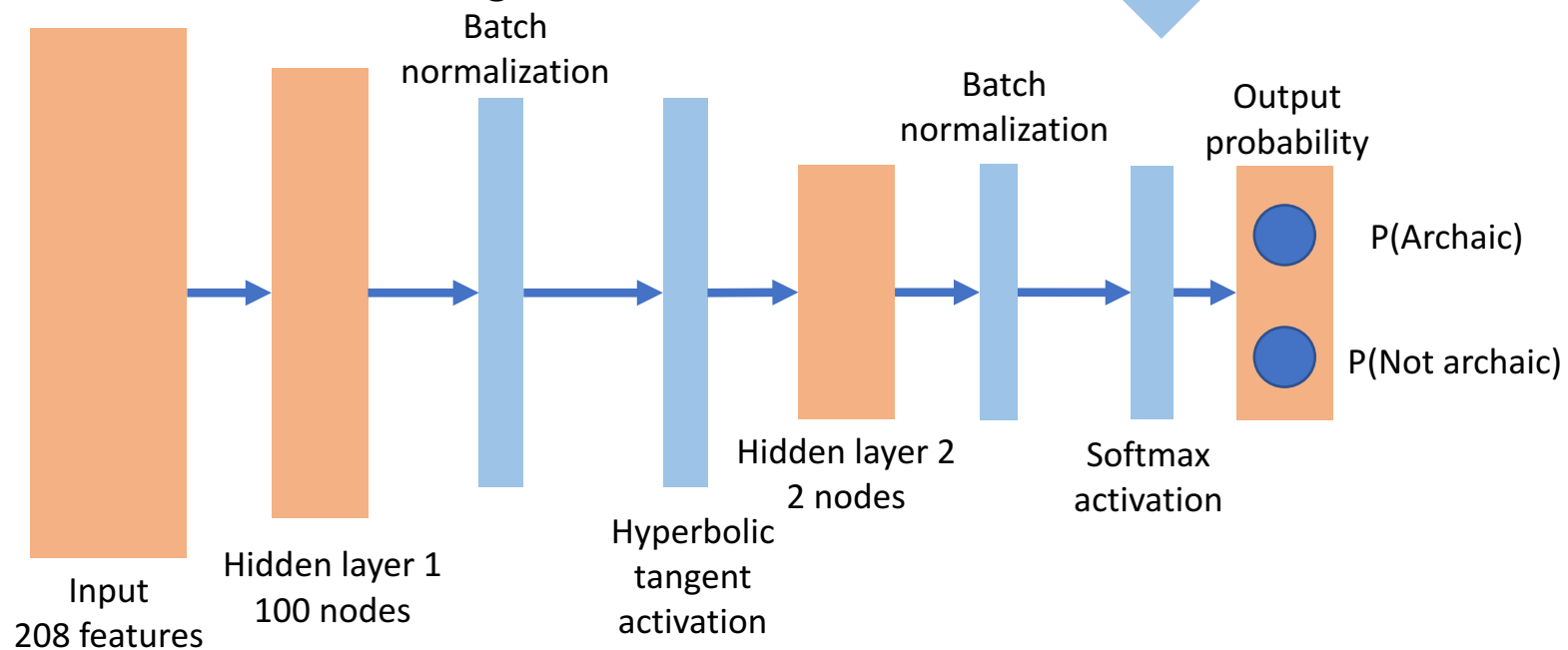

Supplement: S7 Fig — (PDF) [file pgen.1008175.s007.pdf]

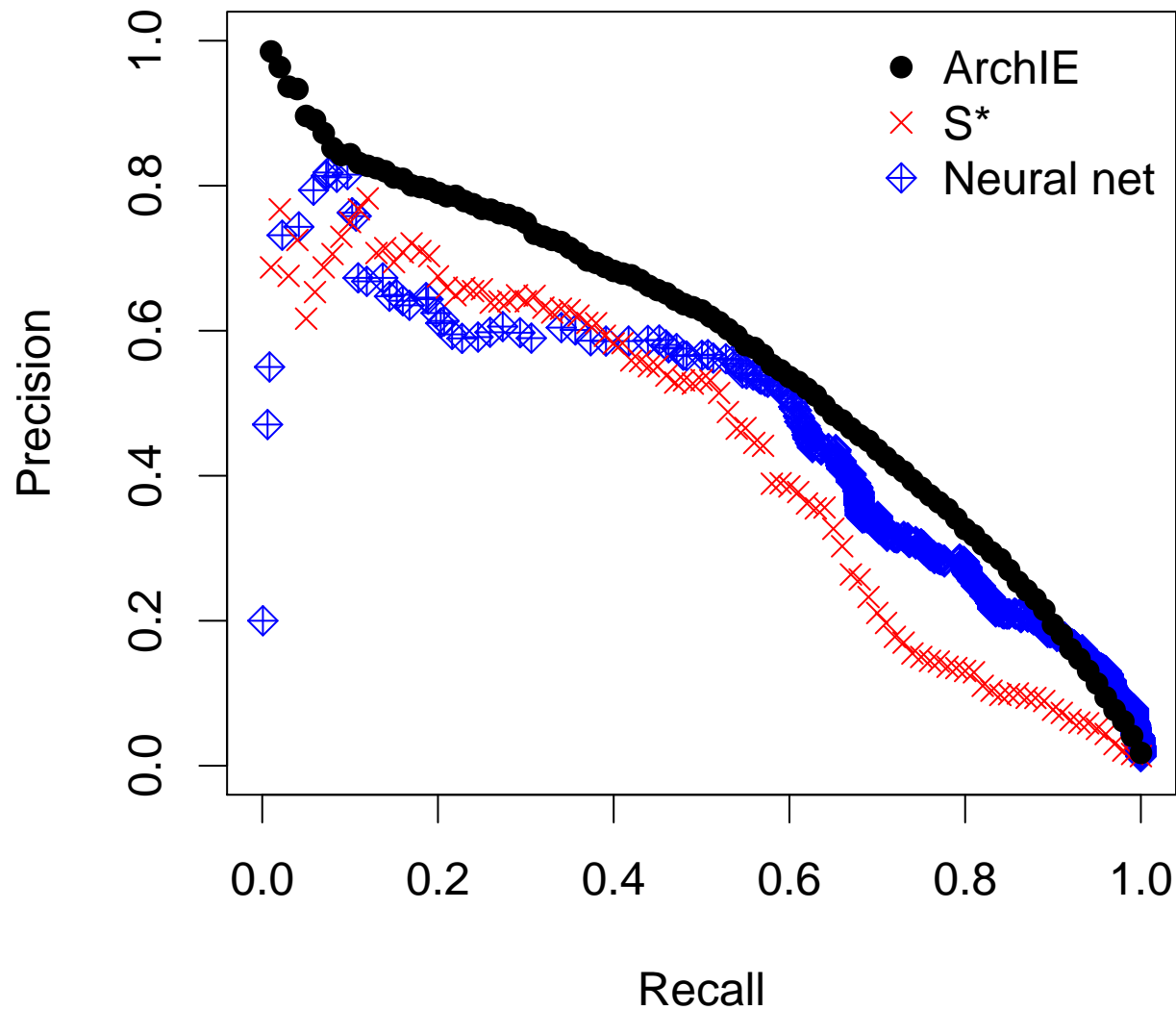

Supplement: S8 Fig — Precision-recall curves for a 2% admixture scenario. Performance of the neural network is shown in blue. (PDF) [file pgen.1008175.s008.pdf]

Altai Neanderthal Match

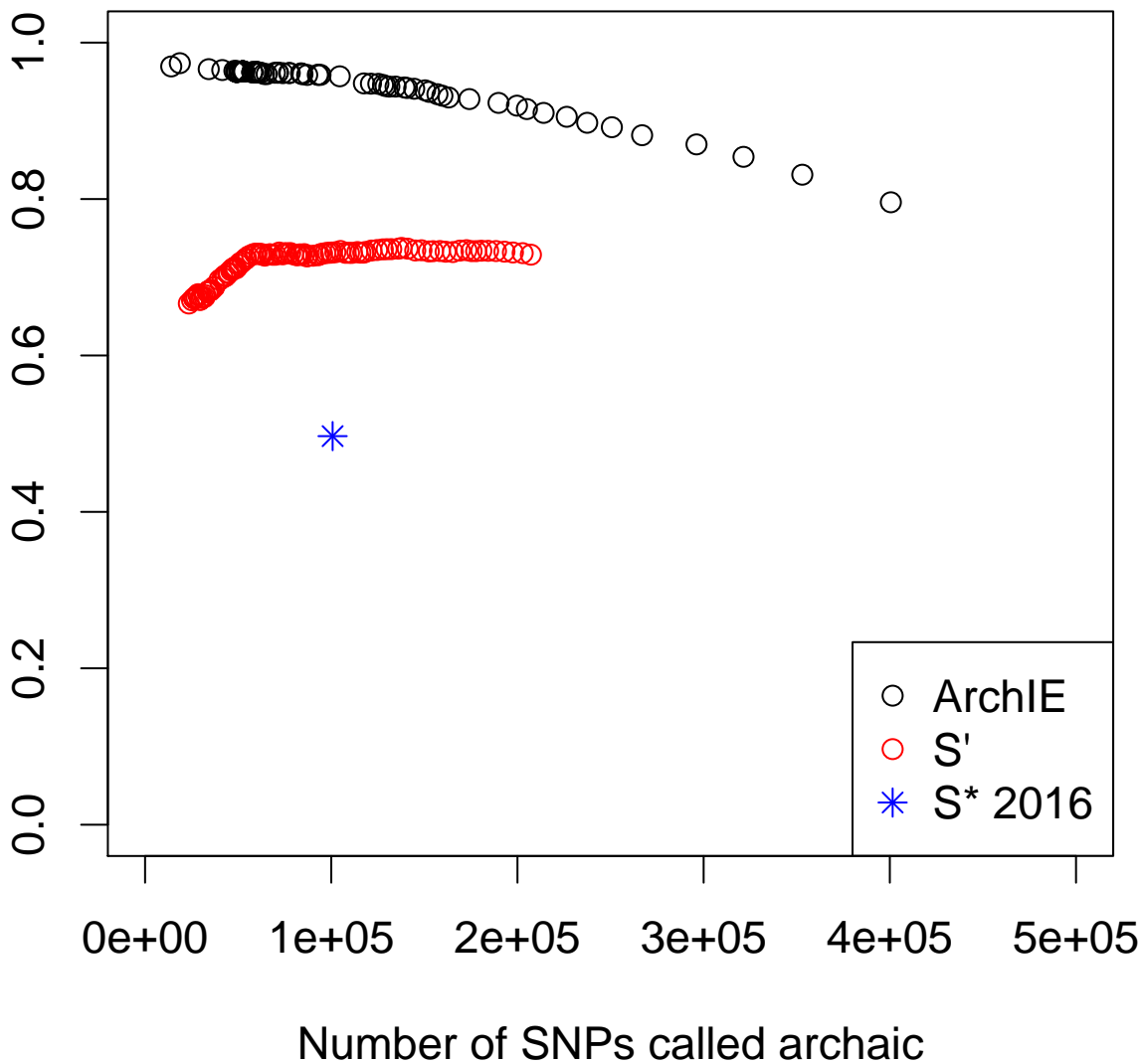

Supplement: S9 Fig — (PDF) [file pgen.1008175.s009.pdf]
