# Supplementary material for: A statistical model for reference-free inference of archaic local ancestry
Source: PLoS Genet. 2019 May 28;15(5):e1008175. doi: 10.1371/journal.pgen.1008175 (PMC6555542; doi:10.1371/journal.pgen.1008175)
Supplement: S1 Text — (PDF) [file pgen.1008175.s011.pdf]

Supplementary materials for ‘A statistical model for reference-free inference of archaic local ancestry’

S1 Neural network model

In addition to the logistic regression model presented in the main text, we applied a neural network model to attempt to learn a function mapping the input features to a binary output [1].

The network architecture consisted of two hidden layers with a hyperbolic tangent non-linear activation function as well as batch normalization [2] between the layers (S7 Fig). There is a final softmax activation layer which outputs the probability of a haplotype containing archaic ancestry ($P(archaic)$) and the probability of no admixture ($P(notarchaic) = 1 - P(archaic)$). To train the network, we used the Adam optimizer, which is an optimizer based on stochastic gradient descent [3].

We tested the performance of the network for the Neanderthal-human demography discussed in the main text and found that while it outperformed the rank based S* algorithm, it did not outperform logistic regression, suggesting that the added non-linearities and features learned from the data do not increase performance over the linear model (S8 Fig).

S2 Comparison of ArchIE with S’ and S* in 1000G data

We compared calls from ArchIE, S’, and S* on 99 CEU individuals from Phase 3 of the 1000 Genomes project.

We obtained S’ calls for alleles with a score of 150,000 or larger as well as a determination of whether the putative archaic allele at a given SNP matches the Altai Neanderthal genotype. We followed Browning *et al.* 2018 and computed the Neanderthal match rate as the number of SNPs with a S’ score above a threshold that match the Altai Neanderthal allele divided by the number of SNPs that are above that threshold and that have a Neanderthal allele that passes filters (marked as either a match or mismatch in the S’ calls). We varied the threshold from 150,000 to 700,000 for S’ and plot the Neanderthal match rate as a function of the number of SNPs called archaic (S9 Fig). At the default S’ threshold of $150,000$, the match rate is 0.73 with a detection rate of around consistent with the results from Browning *et al.*, 2018. We confirmed that the match rate for S’ does decrease slightly at a more stringent threshold. One reason that the match rate might decrease is that there is a decrease both in the numerator and the denominator of the match rate leading to a decrease in the ratio.

We downloaded the S* calls in the 1000 Genomes European individuals released in Vernot *et al.* 2016 and restricted our analysis to CEU individuals. We computed a match rate with the Altai Neanderthal as the number of SNPs within the regions identified in the S* callset that match the Altai Neanderthal divided by the number of variable sites within these regions (following the authors’ definition of Neanderthal matching). We computed the number of SNPs called archaic by determining the number of sites that are derived and not polymorphic (*i.e.* allele frequency is 0 or 1) in a sample of 108 YRI genomes that fall within regions called archaic. Since the S* score associated with each region was not included with the release, we only plot a single point in S9 Fig.

ArchIE computes the probability that an individual haploid genome within a given window is archaic which we then converted into an archaic introgression score for the derived allele at each SNP. We used 50 kb non-overlapping windows and computed, for each SNP in the 50 kb window, the average archaic probability for individuals that carry the derived allele. For example, at chromosome 1, position 51762 there are 4 haploid genomes that carry the derived allele:

- Individual NA12287, haplotype 2
- Individual NA12751, haplotype 2
- Individual NA12761, haplotype 1
- Individual NA12777, haplotype 1

We compute the average probability of archaic ancestry predicted for these four haploid genomes in the window surrounding this SNP.

To ensure comparable results to the S' calls, we followed the procedure outlined in Browning *et al.* 2018 and restricted our analysis to SNPs that pass filters as determined by the Altai Neanderthal genome mask released at `http://cdna.eva.mpg.de/neandertal/Vindija/FilterBed/Altai/`. For a fixed threshold t, we computed the number of SNPs with scores above t as well as the proportion of SNPs with scores above t at which the Altai Neanderthal genome carries a derived allele. These results are reported in S9 Fig for values of $t \in [0, 1]$.

References

[1] Yann LeCun, Yoshua Bengio, and Geoffrey Hinton. Deep learning. *Nature*, 521(7553):436–444, May 2015.

[2] Sergey Ioffe and Christian Szegedy. Batch Normalization: Accelerating Deep Network Training by Reducing Internal Covariate Shift. *arXiv:1502.03167 [cs]*, February 2015. arXiv: 1502.03167.

[3] Diederik P. Kingma and Jimmy Ba. Adam: A Method for Stochastic Optimization. *arXiv:1412.6980 [cs]*, December 2014. arXiv: 1412.6980.
